# Evidence of the importance of contact tracing in fighting COVID-19

**DOI:** 10.4178/epih.e2022006

**Published:** 2022-01-03

**Authors:** Okyu Kwon

**Affiliations:** National Institute for Mathematical Sciences, Daejeon, Korea

**Keywords:** COVID-19, Contact tracing, Epidemics

## Abstract

**OBJECTIVES:**

We analyzed data to determine whether there are distinguishing characteristics depending on the success or failure of control for coronavirus disease 2019 (COVID-19) by country in the trend of the daily number of confirmed cases and the number of tests.

**METHODS:**

We obtained the number of confirmed cases and tests per day for almost every country in the world from Our World in Data. The Pearson correlation between the two time series was calculated according to the time delay to analyze the relationship between the number of tests and the number of cases with a lag.

**RESULTS:**

For each country, we obtained the time lag that makes the maximum correlation between the number of confirmed cases and the number of tests for COVID-19. It can be seen that countries whose time lag making maximum correlation lies in a special section between about 15 days and 20 days are generally been successful in controlling COVID-19. That section looks like a trench on the battlefield.

**CONCLUSIONS:**

We have seen the possibility that the success in mitigating COVID-19 can be expressed as a simple indicator of the time lag of the correlation between confirmed cases and tests. This time lag indicator is presumably reflected by efforts to actively trace the infected persons.

## INTRODUCTION

The coronavirus disease 2019 (COVID-19) epidemic, which started in China at the end of 2019, is still a pandemic as of April 2021. The infectious disease, called COVID-19, has been progressing for a long time and has killed many people, and has caused serious economic damage due to the reduction of social and economic activities. In many parts of the world, interventions to control COVID-19 are being implemented, focusing on quarantine of confirmed cases, social distancing, prohibition of gatherings, and closure of schools. However, controlling infectious diseases is not easy because no one knows exactly who is infected, who is not infected, or who has immunity to the infection. All we know is the infection status of those who have been tested. If an infected person, especially one who has mild symptoms or is asymptomatic, has not been tested, he or she will continue to spread the infection to others. Therefore, in order to control COVID-19, social distancing or prohibitions of gatherings have inevitably been applied to the majority of the population, whose infection status is undetermined. This incurs enormous socioeconomic costs. For this reason, finding infected people quickly through testing is of paramount importance in the fight against COVID-19 [1,2]. The Director-General of the World Health Organization also emphasized the importance of testing by saying that “You cannot fight a fire blindfolded. And we cannot stop this pandemic if we don’t know who is infected” [3]. Indeed, it seems that Korea and Singapore have minimized both the human and economic costs of COVID-19 by identifying who has been infected through extensive screening tests in the early stages of the outbreak. Through the testing, these countries were able to avoid the massive socioeconomic turmoil experienced in Europe and North America by detecting and quarantining infected people, even mild and asymptomatic cases, and by tracking and examining contacts related to confirmed patients.

Claims and studies which emphasize the importance of finding infected individuals through testing and tracking contacts have been introduced [4-6]. Most research on this issue has shown examples for specific countries or demonstrated the importance of these measures through simulations using computational models. We obtained time series data on the number of confirmed cases and the number of tests in almost all countries around the world and analyzed the correlation between both time series according to the time delay. As a result, we found circumstantial evidence indirectly showing that testing and tracking of contacts are effective in suppressing the spread of COVID-19. It was confirmed that countries where the number of confirmed cases peaked and then the number of tests peaked after a certain delay of 2-3 weeks showed successful results in controlling COVID-19. The purpose of this study is to show that testing and contact tracking are effective in fighting the pandemic, not from simulations using imaginary or computational models, but from various actual data in various countries.

## MATERIALS AND METHODS

Our World in Data is an online scientific publication focusing on large-scale global issues such as poverty, disease, hunger, climate change, war, and inequality. In 2020, Our World in Data became one of the leading organizations publishing global data and related research on the COVID-19 pandemic [7]. It creates and maintains a worldwide database on COVID-19 that is used by the United Nations, the White House, the World Health Organization, epidemiologists, and researchers.

**Figure 1.**
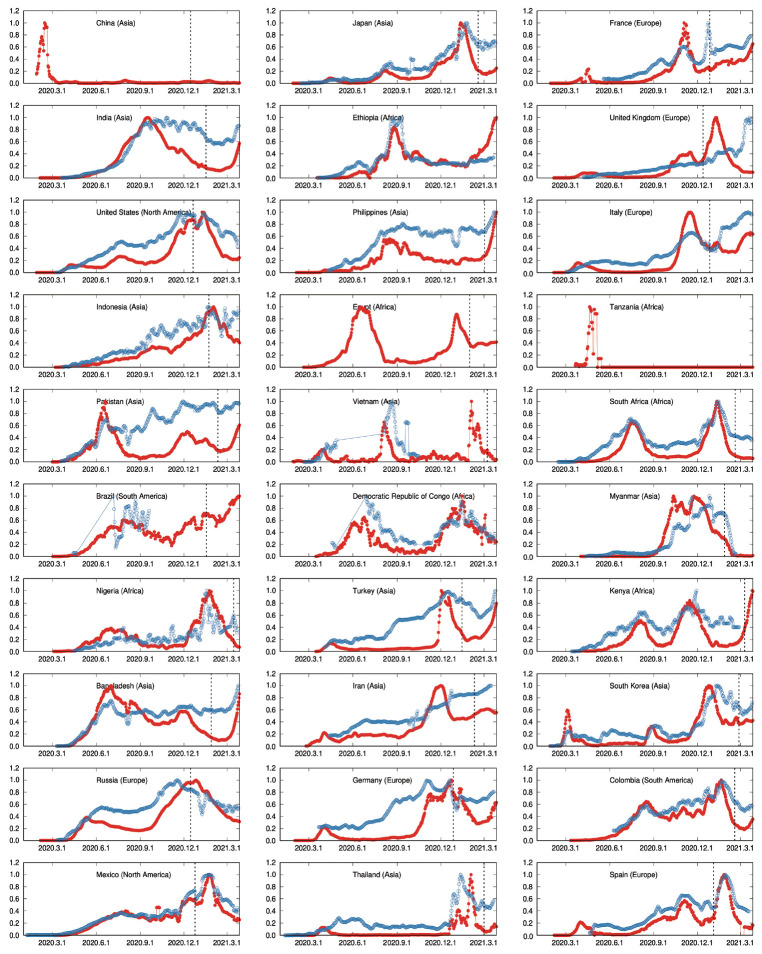
The smoothed daily numbers of new confirmed cases (red) and tests (blue) from January 1 to March 27, 2021. The vertical dashed line separates the periods before and after vaccination programs were launched. No test data exist for China, Egypt, and Tanzania. Brazil, Vietnam, and Democratic Republic of Congo do not have complete data about tests.

From Our World in Data, we obtained the number of cases and tests per day for almost every country in the world. Not only their daily report values but also smoothed time series are provided. Figure 1 shows a graph of the number of cases and the number of tests for the 30 representative countries selected based on the population. The number of tests is usually much higher than the number of confirmed cases. Therefore, the graph was drawn by normalizing the two trends to the largest value in the period we observed to make it easier to see and compare with the eye. Table 1 shows the maximum number of confirmed cases and tests by country. Data on confirmed cases were almost completely recorded in all countries. However, there were some countries where the test data were completely missing or partially missing. China and Egypt had no test data whatsoever, and test data were sparsely missing for Brazil and Vietnam. It was judged that there was a big difference in the environment of the spread of infectious diseases between before and after vaccination was started, and the date when vaccination was first launched in each country is indicated on its graph. The start date differs from country to country, and some countries had not started vaccination during the period we have observed.

### Ethics statement

Not applicable as the manuscript did not involve any experimentation and personal information.

## RESULTS

In Figure 1, it can be seen that the number of confirmed cases per day and the number of tests per day are generally moved in a similar trend. It can be simply understood that the number of confirmed cases increases in proportion to the number of tests. In particular, in the United States, South Africa, Japan, Colombia, Kenya, and Mexico, the trends in the number of tests and the number of confirmed cases seemed to be almost the same. Another feature that can be visually observed is that the number of tests peaked a period of time after the number of confirmed cases peaked. Korea seems to be a representative example of this pattern. Another pattern is that the number of tests continued to be high even after the number of confirmed cases peaked, as observed in countries such as India and the Philippines. This finding can be interpreted as indicating that if the number of tests is maintained even after the number of confirmed cases has peaked, the spread of the infection is suppressed and the number of confirmed cases gradually decreases because people with covert infections continue to be discovered. Still another pattern is exemplified by Russia, where the number of tests peaked first, and then the number of confirmed cases peaked later. An explanation for this pattern is that if testing is reduced at an early stage, the spread of the infection continues by people with undetected infections, and the number of confirmed cases later increases. The time period of the analyzed data varied from country to country and all results are obtained by using only data up to the day before the start of vaccination.

### Correlation with time lag

In order to systematically analyze the relationship between the number of tests and the number of cases with a lag, the Pearson correlation between the two time series was calculated according to the time delay. We obtained the Pearson correlation *CORR_x_*(*τ*) as a function of the time delay *τ*∈[-40,40]. The functional form can be written as


(1) $$CORR_{x}(\tau )\;=\;\frac{\sum_{i=\tau }^{L_{x}}(C_{x}(1-\tau )-\bar{C_{x}})(T_{x}(i)-\bar{T_{x}})}{\sqrt{\sum_{i=\tau }^{L_{x}}(C_{x}(i-\tau )\bar{C_{x}}{)}^{2}\sqrt{\sum_{i=\tau }^{L_{x}}(T_{x}(i)-\bar{T_{x}}{)}^{2}}}},\;\bar{C_{x}}=\frac{\sum_{i=\tau }^{L_{x}}C_{x}(i-\tau )}{\left(L_{x}+1-\tau \right)},\;\bar{T_{x}}=\frac{\sum_{i=\tau }^{L_{x}}T_{x}\left(i-\tau \right)}{\left(L_{x}+1-\tau \right)},\;\;$$

Where *C_x_*(*i*) and *T_x_*(*i*) are the number of confirmed cases and tests on the day *i* in country *x*, respectively, and *L_x_* is the date length of the data used for country *x*. It was thought that the situation regarding the spread of the infection significantly changed after the start of vaccination programs; therefore, only the period before vaccines were introduced into a country was considered. This method can be easily intuitively understood as calculating the correlation between the number of confirmed cases on day *i* and the number of tests after *τ* days from after day *i*. Through this calculation, the time difference $\tau_{x}^{max}=argmax(CORR_{x}(\tau ))$, that produced the maximum correlation, was obtained for each country.

**Figure 2.**
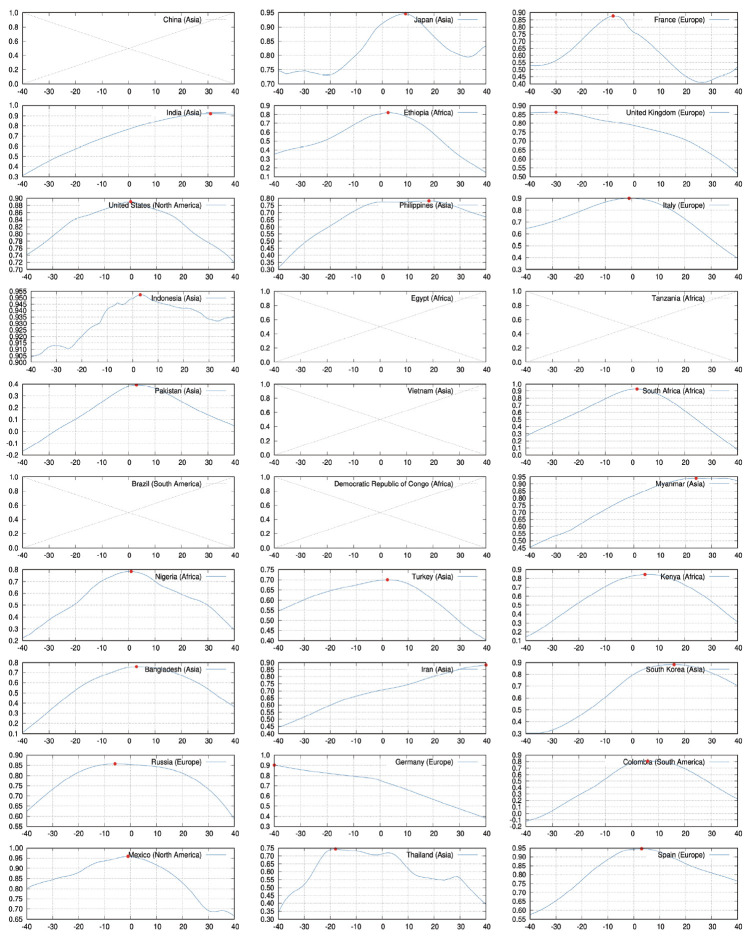
The correlation coefficient according to the time lag between the number of confirmed cases and the number of tests. The *x*-axis is the time difference and has the unit of days. The *y*-axis is the correlation coefficient value. The location with the largest correlation coefficient value is indicated by a red dot.

Figure 2 shows the results of the correlation analysis, excluding 6 countries that did not have complete test data from the top 30 countries according to population. In general, the graph shows the shape of a mountain with a single peak, and the lag value of $\tau_{x}^{max}$ at which the peak is located differs from country to country. Most are around $\tau_{x}^{max}$ = 0, and there are seldom cases where $\tau_{x}^{max}$ is outside the range of ± 10 days. In many countries, the number of confirmed cases and tests are showing a similar trend without a large time lag.

For Korea, which is considered to be a country that successfully controlled COVID-19, the $\tau_{x}^{max}$ was calculated as 16 days. This reflects the trend of the time series in Figure 1, where the number of test peaked 16 days after the number of confirmed cases peaked.

In contrast, for the United States, which is evaluated as unsuccessful in controlling COVID-19, the $\tau_{x}^{max}$ was 0. This is clearly shown by the graph in Figure 1, where the number of confirmed patients and the number of tests exhibit the same trend without a lag.

### The time lag as a possible indicator of success degree for fighting COVID-19

**Figure 3.**
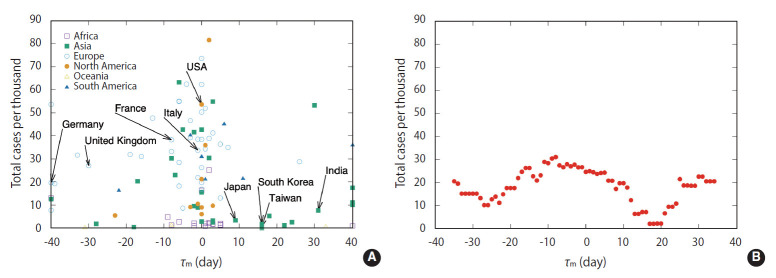
(A) Each point in represents a country, the *x*-axis is the time lag value at which the correlation of the country is maximized, and the *y*-axis is the total number of confirmed cases per thousand population. There are 106 points. Each point is marked by different colors and shapes for each continent to which each country belongs. (B) Shows the average of the total number of confirmed cases per thousand population for all countries included in the 11-day window of the *x*-axis.

Focusing on the results of the above correlation analysis, we examined the possibility that the time lag between the peak in confirmed cases and the peak in tests could be an index to evaluate the success of COVID-19 control. For all countries with complete data on the number of confirmed cases and the number of tests, the time lag value ($\tau_{x}^{max}$) with the greatest correlation between them was calculated. We considered the total cumulative number of confirmed cases per 1,000 population as a measure of degree of a country’s success in fighting COVID-19. To see the relationship between the time lag and the success, a scatter plot between $\tau_{x}^{max}$ and total cases per thousand was drawn as shown in Figure 3A. It can be seen that many countries are clustered near $\tau_{x}^{max}$ = 0, where the number of confirmed cases relative to the population is widely distributed from small to many cases. On the other hand, countries whose $\tau_{x}^{max}$ is outside the range of -10 days and 10 days tended to have relatively few confirmed cases per population. In particular, countries where $\tau_{x}^{max}$ was between the 15 days and the 20 days showed a remarkably low number of confirmed cases compared to the population. Korea, Taiwan, and Japan were located in that range. To see this trend more clearly, the average of the *y*-axis values for all the countries included in the window of 11 days length along the *x*-axis in Figure 3A was calculated. The result can be seen in Figure 3B, and it can be seen that a deep valley appears between 15 days and 20 days of $\tau_{x}^{max}$. The pattern where the number of tests peaked 2-3 weeks later after the number of confirmed cases peaked can be understood as reflecting the active search for targets to be tested through contact tracing. As the number of confirmed cases increases, the number of related contacts increases, and tests for them are followed. Therefore, the number of tests does not decrease immediately but increases and then decreases with a lag.

In Figure 3A, the features of the country’s location can be seen divided by continent. In most European and North American countries, the time lag $\tau_{x}^{max}$ was near zero or had a negative value. This category included many countries with a large number of infected people. An interpretation of this finding can be assumed that European and North American countries that value personal freedom had challenges in contact tracing due to difficulties in handling personal information. On the other hand, in many Asian countries, especially Korea, Taiwan, and Japan, the time lag had a positive value and the number of infections was low. In Korea, after the spread of Middle East respiratory syndrome in 2017, it became legally possible to handle personal information in an infectious disease outbreak. Accordingly, it is possible to track contacts of confirmed cases using information such as mobile phones and credit cards. Asian countries where these measures are possible exhibit the characteristic of generating positive values of $\tau_{x}^{max}$ as are generated as a result of active contact tracing. Most countries in the African showed a time lag near zero, as in Europe and North America. However, the number of infected people remained fairly low. Human migration between continents or regions is relatively low compared to countries on other continents, so the spread of infectious diseases may not be significant.

## DISCUSSION

Through the analysis of the time-lagged correlation between the number of confirmed cases and the number of tests, we found evidence supporting the inference that detecting additional infected cases through contact tracing is an important factor in the fight against COVID-19. An analysis of empirical data confirmed that nations wherein the number of tests peaked 2-3 weeks later after the peak number of confirmed cases effectively controlled the spread of COVID-19. The reason for the lag of 2-3 weeks is most likely that testing was actively performed to confirm the spread of the infection by tracing the contacts of confirmed cases. These efforts are believed to be of great help in controlling the spread of COVID-19.

Obviously, some countries had very low rates of confirmed cases, even where $\tau_{x}^{max}$ was outside the 15-day to 20-day range. Contact tracing is not the only method of responding to the COVID-19 pandemic. Through containment and strong social distancing, contact between people can be reduced. In addition, thorough hygiene management such as mask-wearing and hand-washing can reduce the likelihood of infection during contact. It is possible that the spread of COVID-19 was suppressed in some countries regardless of the value of $\tau_{x}^{max}$ by these various mitigation strategies.

The fact that countries with a lag of 15-20 days, or 2-3 weeks, were successful in fighting against COVID-19 may be a noteworthy result reflecting the principle of contact tracking and the incubation period characteristics of infectious diseases. These results may have important implications regarding the optimal strategy for effective contact tracing and for carrying out inspections accordingly. It will be a meaningful study for future research to investigate why this particular time lag occurs through a model simulating infectious disease spread that incorporates the mechanism of contact tracing.

## Figures and Tables

**Table 1.** The maximum number of confirmed cases per day and the maximum number of daily tests for the period from January 1 to March 27, 2021 in the top 30 countries by population size

Continent	Country	Maximum cases	Maximum tests
Asia	China	4,607	-
Asia	India	93,199	1,196,972
North America	United States	250,744	1,864,725
Asia	Indonesia	12,865	49,183
Asia	Pakistan	6,533	42,212
South America	Brazil	77,129	98,318
Africa	Nigeria	1,666	19,768
Asia	Bangladesh	3,810	23,648
Europe	Russia	28,501	565,313
North America	Mexico	17,559	37,414
Asia	Japan	6,446	74,090
Africa	Ethiopia	1,879	21,880
Asia	Philippines	8,055	47,614
Africa	Egypt	1,575	-
Asia	Vietnam	56	24,857
Africa	Democratic Republic of Congo	249	869
Asia	Turkey	33,307	203,635
Asia	Iran	13,630	63,195
Europe	Germany	25,757	238,862
Asia	Thailand	943	30,718
Europe	France	56,225	515,517
Europe	United Kingdom	59,829	1,409,016
Europe	Italy	35,073	329,462
Africa	Tanzania	29	-
Africa	South Africa	19,042	66,585
Asia	Myanmar	1,505	26,928
Africa	Kenya	1,327	8,890
Asia	South Korea	1,047	54,529
South America	Colombia	17,857	73,144
Europe	Spain	37,011	257,295
